# Supplementary material for: Catatonic Features That May Be Mistaken for Worsening Autism Show Treatment-Associated Improvement: Implications for Later Regression
Source: medRxiv. 2026 Sep 15:2026.09.14.26363007. Preprint. [Version 1] doi: 10.64898/2026.09.14.26363007 (PMC13596598; doi:10.64898/2026.09.14.26363007)

Supplemental Figure 1: Autism-Specific Kanner Item-Level Change

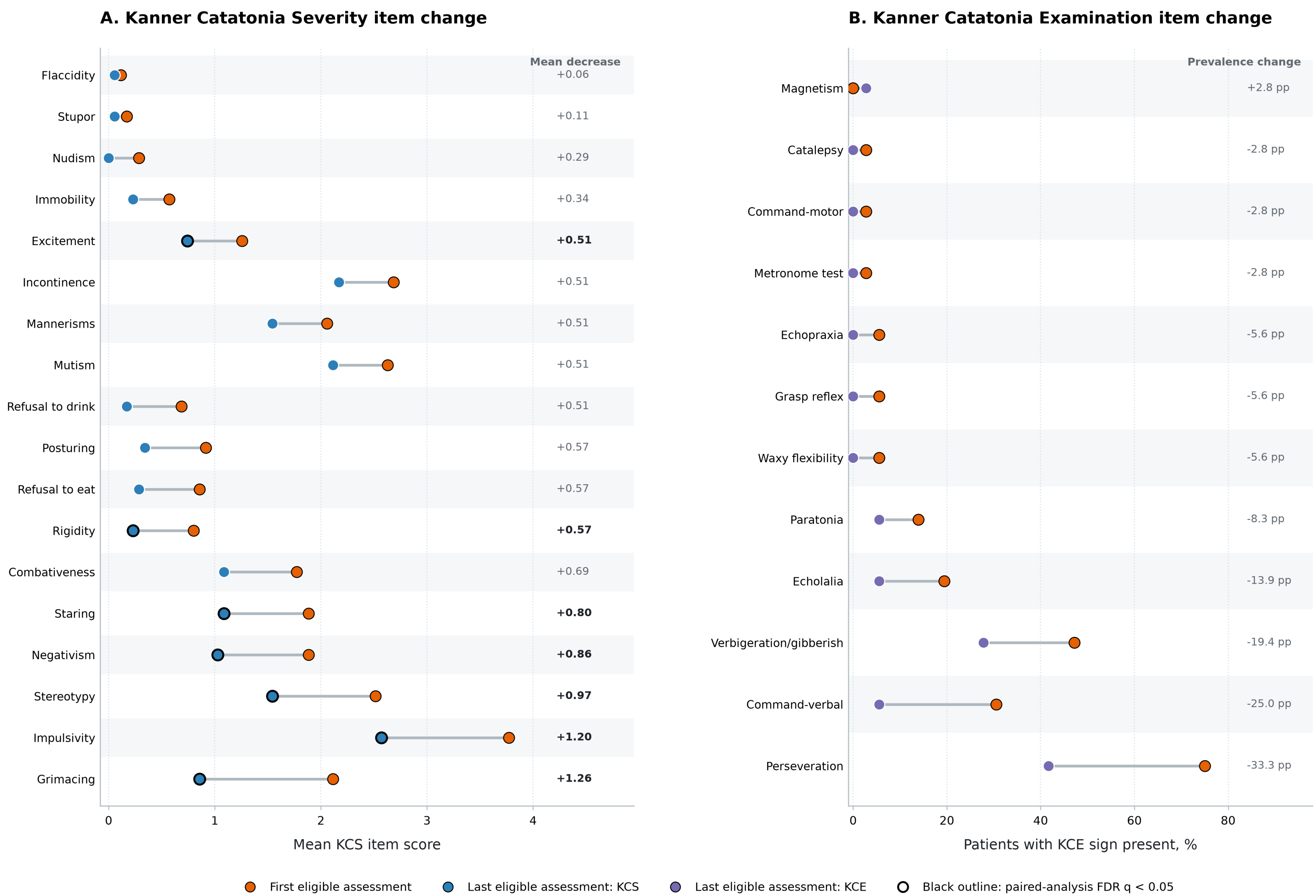

Supplement: Supplementary file 1 [file media-1.pdf]
